# Association between patent ductus arteriosus flow and home oxygen therapy in extremely preterm infants

**DOI:** 10.1038/s41390-024-03120-8

**Published:** 2024-03-07

**Authors:** Jana Termerova, Ales A. Kubena, Karel Liska, Viktor Tomek, Richard Plavka

**Affiliations:** 1 Department of Gynecology, Obstetrics and Neonatology, First Faculty of Medicine, Charles University and General University Hospital in Prague, Prague, Czech Republic; 2 Institute of Medical Biochemistry and Laboratory Diagnostics, First Faculty of Medicine, Charles University and General University Hospital in Prague, Prague, Czech Republic; 3 Children’s Heart Center, Second Faculty of Medicine, Charles University and Motol University Hospital in Prague, Prague, Czech Republic

## Abstract

**Background:**

Central blood flow measurements include the estimation of right and left ventricular output (RVO, LVO), superior vena cava (SVC) flow, and calculated patent ductus arteriosus (PDA) flow. We aimed to provide an overview of the maturation patterns of these values and the relationship between PDA flow and the need for home oxygen therapy.

**Methods:**

This prospective single-center study was conducted in infants born at <26 weeks of gestation. We performed echocardiographic measurements five times during their life (from the 4th post-natal day to the 36th postmenstrual week).

**Results:**

Sixty patients with a mean birth weight of 680 (590, 760) g were included. Postnatal development of LVO and PDA flow peaked at the end of the second postnatal week (427 and 66 mL/kg/min, respectively). The RVO increased between days 4 and 7–8. The SVCF was most stable. The development curves of PDA flow differed between the groups with (*n* = 28; 47%) and without home oxygen therapy.

**Conclusion:**

We present the central blood flow values and their postnatal development in infants <26 weeks of gestation. This study demonstrates the association between PDA flow and the future need for home oxygen therapy.

**Impact:**

- This study enriches our knowledge of the long-term development of central blood flow parameters and derived patent ductus arteriosus (PDA) flow in extremely preterm infants (<26 weeks).
- While pulmonary resistance decreased, PDA flow continued to increase from day 4 to the end of the second week of life. Similarly, left ventricular output increased as a marker of preload. The superior vena cava flow remained stable.
- The observed association between PDA flow and an unfavorable respiratory outcome is important for future studies focusing on the prevention of chronic lung disease.

## Introduction

Extremely premature neonates, especially those born before 26 weeks of gestation, are a special and particularly vulnerable group of infants. Their immature myocardium must rapidly adapt to the high systemic vascular resistance after birth. Their pulmonary blood flow can be increased by a persistent left-to-right shunts. Using central blood flow (CBF) measurements, we can estimate the cardiac output from the right and left ventricles and systemic return from the upper half of the body based on superior vena cava (SVC) flow. Because CBF values are more valuable than blood pressure for assessing systemic perfusion^1^, neonatologists are increasingly using these functional echocardiographic variables to evaluate various pathological conditions in real time, most commonly sepsis, or to assess the hemodynamic significance of a patent ductus arteriosus (PDA). CBF measurements reflect global cardiac function (preload, contractility, and afterload) and are probably the most informative if followed over time^2^.

To the best of our knowledge, a comprehensive overview of the postnatal evolution of CBF values has not been established for extremely preterm neonates born at less than 26 weeks of gestation. Clinical studies have shown that low SVC flow is associated with late intraventricular haemorrhages (IVH)^3^ and abnormal neurodevelopmental outcomes^4^. In the studies published to date, central flows have been investigated mainly in the early transitional period (usually in the first three days of life). The process of adaptation to extrauterine life takes much longer. Exposure to left-to-right ductal shunts in extremely immature infants may take several weeks, and lung maturation takes even longer. Therefore, in our study, we focused on the development of CBF from the 4th postnatal day to the 36th postmenstrual week. CBF values in stable preterm infants after the transition period, but only at 7 and 14 days of life, were studied by Sloot et al.^5^. Their study population included more mature infants (mean age 28 weeks of gestation) and only infants with a PDA diameter <1.4 mm.

An increased risk of developing chronic lung disease is another characteristic of immature infants younger than 26 weeks of gestation. Their lung development from the canalicular stage onward occurs in an unnatural environment, in which the pulmonary vasculature is often subjected to a prolonged left-to-right shunt. This may interfere with the normal maturation process during the development of pulmonary circulation. However, the role of high pulmonary flow rate and its duration in the development of chronic lung disease is still not elucidated. Many interventional studies have been conducted on the treatment of PDA;^6,7^ however very few observational studies have assessed the duration and amount of PDA blood flow.

The primary objective of this study was to provide a comprehensive overview of the development of CBF values in extremely preterm infants during postnatal life. A secondary objective was to compare the evolution of CBF values and derived PDA flow between the group that developed a need for home oxygen therapy and the group that was discharged home without oxygen requirement. The results of this observational study may clarify the possibility of adaptation of the immature myocardium to extrauterine life and the impact of PDA on the development of chronic lung disease.

## Methods

This was a prospective, single-center, longitudinal, observational cohort study conducted between 9/1/2018–10/31/2022 in the tertiary neonatal intensive care unit of Charles University and General University Hospital in Prague. This study was approved by the Ethics Committee of General University Hospital in Prague (document ID 17/21 S- IV). The parents of all eligible infants were provided with an informational sheet and were fully informed, after which written informed consent was obtained.

### Patients and protocol

Immature infants born before 26 weeks of gestation were followed until discharge to home care. Infants with congenital anomalies (other than PDA) or severe multi-organ failure were excluded. Infants who received ventilatory or cardiac support were included in the study, and these supports were recorded in detail. Infants who died at < 36 weeks of postmenstrual age (PMA) during the study period were excluded from the primary analysis. Basic birth and clinical data were collected, including weight, postnatal steroid administration, PDA treatment, duration of respiratory support, and neonatal morbidities such as IVH according to the Papile classification^8^, bronchopulmonary dysplasia (BPD) according to the Jensen classification^9^ assessed at 36 weeks of PMA, retinopathy of prematurity (ROP) and necrotizing enterocolitis (NEC) with radiological evidence of pneumatosis intestinalis.

Prospective echocardiographic examinations were performed at 5 time points: day 4 (72–96 h), day 7–8, day 14–15; and at 30 and 36 weeks of PMA. Criteria for home oxygen therapy after discharge were: infants unable to maintain oxygen saturation >96%. Peri-viable infants with a high-risk score for IVH of > 38% were prophylactically treated with indomethacin (Liometacen, Chiesi)^10^. PDA in infants with signs of cardiac impairment (mitral regurgitation, oliguria, and respiratory failure) received a later treatment - ibuprofen (Pedea, Recordati Rare Disease, France) according to clinical decisions. The scheme of the study protocol was continued by measurement of the CBF, whether the treatment of PDA was successful or not. The success of the pharmacological treatment was reflected in the CBF values.

### The methodology of Doppler-derived CBF measurements

All echocardiographic images were acquired using a Vivid 7 ultrasound machine (GE Healthcare, Milwaukee, WI) with a 10-MHz sector probe by a single experienced investigator (J.T.), the values were calculated at the bedside. Echocardiographic assessment of blood flow shows a high inter- and intra-observer variability and margin of error. Inter-observer variability was minimized in this study because the scans were performed by a single examiner.

At each examination, congenital heart disease was excluded, and the following comprehensive functional echocardiographic measurements were performed.

The cross-sectional area (CSA) of the central vessels was calculated as: *π* × (diameter/2)^2^. Left ventricular output (LVO) was measured as follows: the inner diameter of the ascending aorta was recorded in the parasternal long-axis view at the end of systole, and the velocity-time integral (VTI) of the ascending aorta was measured using pulse-wave Doppler in the apical 5-chamber view (the cursor was aligned with the flow direction; a minimum insonation correction < 20° was used). The LVO (mL/kg/min) was then determined as follows: LVO = aortic CSA × VTI × heart rate/weight in mL/kg/min. The diameter of the right ventricular outflow tract was measured in the parasternal view at the attachment of the pulmonary valve in end-systole, VTI was recorded from the same view as close as possible to the valve, and then right ventricular output (RVO) was calculated as: RVO = pulmonary CSA × VTI × heart rate/weight in mL/kg/min.

According to the original study describing the SVC flow, the mean SVC diameter was measured in a modified parasternal long-axis view at the point where the vessel walls clearly began to open into the right atrium^11^. Pulsed Doppler recordings for VTI tracings were taken from a low subcostal view and in the case of significant differences between individual cycles, an average of 3–5 adjacent cycles was used. SVC flow was calculated as VCS CSA × VTI × heart rate/weight (mL/kg/min). PDA flow was calculated from the measured central flows: LVO-2.7 × SVC flow, because SVC flow accounts for a mean of 37% of the total systemic blood flow^11^. When the PDA was closed, PDA flow was counted as zero. Pulmonary flow was estimated as the sum of the PDA flow and RVO. The diameter of the ductus arteriosus was measured in the high left parasternal view, ideally without colour-flow Doppler imaging. When this was not possible, the scale and gain settings of the colour-flow Doppler were optimized.

### Data analysis

Statistical analyses were performed using SPSS (version 23.0) and Wolfram Mathematika (version 12.1 and 12.3). All data are expressed as medians and interquartile ranges (1–3 interquartiles). The Friedman test was used to evaluate changes in CBF from the fourth postnatal day to 36 weeks of PMA. A general linear repeated-measures model was used to test within- and between-subject main effects of PDA flow and the need for home oxygen therapy. The Bonferroni method allowed pairwise comparisons of means. Statistical differences of baseline clinical data between the two groups of infants were calculated using chi-square tests for categorical variables and Mann–Whitney *U* tests for quantitative variables, with statistical significance set at *P* < 0.05.

## Results

**Fig. 1:**
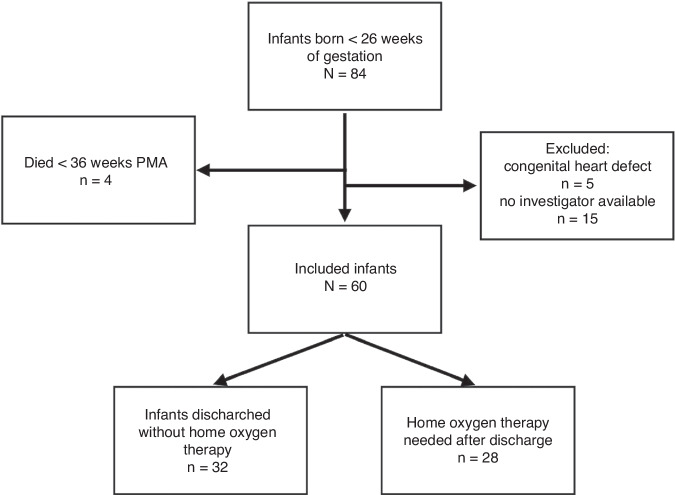
Flowchart analysis. Infant enrolment, mortality and proportion of infant with the need for home oxygen after discharge.

Of the 84 infants born before 26 weeks of gestation who were eligible for the study, 60 from a four-year period were included (Fig. 1: Flowchart). Four infants died before 36 weeks of PMA: three infants died early after birth on day 3–4 (two due to sepsis and one due to severe perinatal asphyxia following placental abruption). Five infants were excluded because of a congenital heart defect (4 × pulmonary stenosis, 1 × ventricular septal defect). Fifteen eligible infants could not be included because the investigator was not available to perform a scan (the covid pandemic interrupted the continuity of the study). In 48 infants, all five echocardiographic examinations were available, in 12 infants, one or two of the five examinations was missing. The clinical data of the mothers and infants are summarized in Table 1. The clinical characteristics of the study population at each time point are presented in Table 2.

**Table 1:** Clinical data of the mothers and infants.

<26 GA	All	Home oxygen Yes	Home oxygen No	*P* value
Total number of infants, *n*	60	28	32	
Birth weight, kg median (IQR)	0.675 (0.59–0.755)	0.65 (0.55–0.74)	0.71 (0.64–0.77)	0.02
Gestational age median (IQR)	24.71 (24.14–25.43)	24.29 (23.71–24.64)	25.29 (24.64–25.57)	<0.01
Sex - male (%)	26 (43)	14 (50)	12 (38)	0.33
Multiples (%)	7 (12)	6 (21)	1 (3)	<0.01
Chorioamnionitis (%)	49 (84)	23 (85)	26 (84)	0.89
Antenatal steroids > 12 h (%)	38 (63)	14 (50)	24 (75)	0.04
1 min Apgar median (IQR)	5 (3–7)	4 (2–6)	6 (3.5–7)	0.02
5 min Apgar median (IQR)	7 (6–8)	6.5 (4.5–8)	8 (6–9)	<0.01

**Table 2:** Clinical characteristics of the study population at each time point of examination.

Timing of ECHO	Day 4	Day 7–8	Day 14–15	30 weeks’ PMA	36 weeks’ PMA
Total infants for analysis, n	57	58	58	57	56
PDA ≥ 1.5 mm, *n* (%)	26 (46)	23 (40)	28 (48)	12 (21)	5 (9)
PDA 0.1–1.5 mm, *n* (%)	11 (19)	11 (19)	8 (14)	7 (12)	7 (13)
PDA closed, *n* (%)	20 (35)	24 (41)	22 (38)	38 (67)	44 (79)
Invasive ventilation, *n* (%)	42 (74)	42 (72)	42 (72)	17 (30)	1 (2)
Noninvasive respiratory support, *n* (%)	15 (26)	16 (28)	16 (28)	40 (70)	41 (73)
No respiratory support, *n* (%)	0 (0)	0 (0)	0 (0)	0 (0)	14 (25)
Mean airway pressure (cm H20), median (IQR)	6.9 (5.5–7.93)	7.2 (6.03–8)	8.41 (7–11)	9.15 (7–11)	not applicable
Cardiac support, *n* (%)	7 (12)	7 (12)	4 (7)	1 (2)	0 (0)

The median (interquartiles) weight at birth was 680 (590–755) g, and the median (interquartiles) gestational ages was 24,71 (24.14-25.43) weeks. Pharmacological treatment for PDA was offered for 40% of the infants, of whom 15% were treated prophylactically with indomethacin to prevent IVH and 25% received a later treatment with ibuprofen. The success rate of the late treatment was very low in both our groups (22% in the group with home oxygen therapy and 25% in the group without home oxygen therapy). The median (interquartiles) of PMA at discharge was 41.43 (39.57–42.75) weeks. The infants who required home oxygen therapy (*n* = 28, 47%) were younger and smaller. They were more often from multiple pregnancies and had received fewer steroids before birth. Many of them received surfactants after birth, spent more days on the ventilator, and required longer noninvasive respiratory support. All were treated with postnatal steroids. The two groups did not differ in the incidence of ROP, IVH, NEC, and sepsis (Table 3).

**Table 3:** The need for treatment and the occurrence of serious complications in the studied population.

<26 GA	All	Home oxygen Yes	Home oxygen No	*P* value
Total number of infants, *n*	60	28	32	
Surfactant administration, *n* (%)	48 (80)	26 (93)	22 (69)	0.02
PDA treatment, *n* (%)	24 (40)	13 (46)	11 (34)	0.34
Prophylactic indomethacin, *n* (%)	9 (15)	4 (14)	5 (16)	0.88
Postnatal steroids, *n* (%)	44 (73)	28 (100)	16 (50)	<0.01
Sepsis, *n* (%)	32 (53)	18 (64)	15 (47)	0.18
NEC ≥ 2b, *n* (%)	10 (17)	4 (14)	6 (19)	0.64
ROP (treated), *n* (%)	8 (13)	4 (14)	4 (13)	0.84
Days on invasive ventilation support, median (IQR)	33 (19–46.5)	42.5 (35.5–53.5)	20 (2.5–33.5)	<0.01
The day of the end of noninvasive ventilation, median (IQR)	89 (75–101.5)	100.5 (91–108)	77.5 (62.5–90.5)	<0.01
BPD grade 0–1/2/3, *n* (%)	26/32/2 (43/53/3)	4/22/2 (14/79/7)	22/10/0 (69/31/0)	<0.01
IVH (grade 3 or 4), *n* (%)	3 (5)	2 (7)	1 (3)	0.48

*BPD* bronchopulmonary dysplasia, *IVH* intraventricular haemorrhages, *NEC* necrotising enterocolitis, *PDA* patent ductus arteriosus, *ROP* retinopathy of prematuriry, *IQR* 1–3 interquartiles.

### The evolution of central blood parameters

**Fig. 2:**
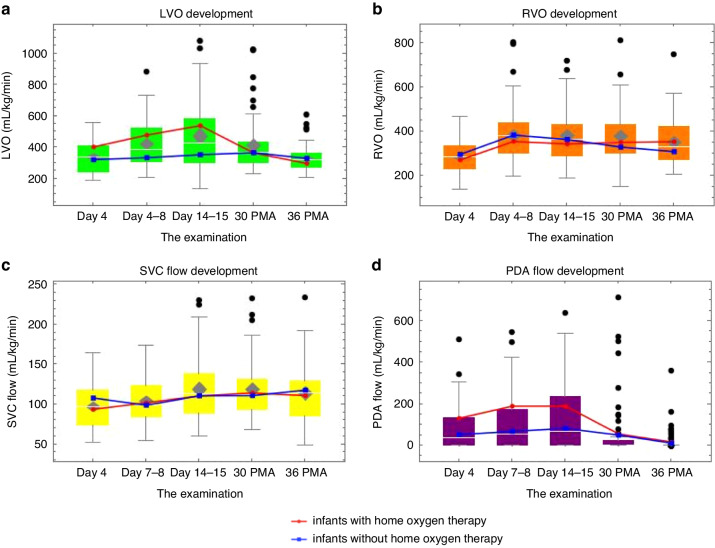
Development of central blood flow. The graph shows **a** left ventricular output development, **b** right ventricular output development, **c** superior vena cava flow development, and **d** patent ductus arteriosus flow development. Colored bars represent the entire population. Blue line children without need for home oxygen therapy and red line children with future need for home oxygen therapy. LVO left ventricular output, PDA patent ductus arteriosus, PMA postmenstrual age, RVO right ventricular outflow, SVC superior vena cava.

All monitored CBF parameters develop over time according to the Friedman test. The trends in the central flow for the entire cohort are summarized in Table 4 and shown graphically in Fig. 2. Table 4 also shows the maturation of the vessel diameters and velocity-time integrals.

**Table 4:** The trends in the central blood flow for the entire cohort.

	Day 4	Day 7–8	Day 14–15	30 weeks’ PMA	36 weeks’ PMA	*P* value (Friedman test)
Total infants for analysis, *n*	56	59	58	57	56	
Weight kg, median (IQR)	0.68 (0.59–0.76)	0.65 (0.58–0.7)	0.71 (0.62–0.79)	1.05 (0.95–1.16)	2.08 (1.92–2.33)	<0.001
LVOd cm, median (IQR)	0.43 (0.41–0.47)	0.44 (0.42–0.47)	0.46 (0.42–0.5)	0.52 (0.49–0.55)	0.65 (0.6–0.7)	<0.001
LVO VTI cm, median (IQR)	8.95 (7.5–10.7)	9.9 (7.7–12.7)	10.6 (8.5–14.3)	10.7 (9–12.13)	12.5 (11.15–13.65)	<0.001
LVO mL/kg/min, median (IQR)	336 (240–406)	384 (305–521)	427 (301–582)	364 (297–429)	316 (266–361)	<0.001
RVOd cm, median (IQR)	0.47 (0.45–0.5)	0.5 (0.46–0.52)	0.5 (0.47–0.54)	0.56 (0.52–0.6)	0.73 (0.7–0.8)	<0.001
RVO VTI cm, median (IQR)	6.5 (5.5–7.8)	7.3 (6–8.9)	7.75 (6–8.9)	8.8 (7.9–9.8)	10.1 (8.6–12)	<0.001
RVO mL/kg/min, median (IQR)	280 (226–330)	376 (297–437)	361 (287–429)	347 (298–427)	327 (268–418)	<0.001
SVCd cm, median (IQR)	0.23 (0.22–0.24)	0.23 (0.22–0.24)	0.24 (0.23–0.25)	0.28 (0.26–0.3)	0.36 (0.32–0.4)	<0.001
SVC VTI cm, median (IQR)	9.8 (8.5–11.1)	9.2 (8.4–11)	10.15 (9.1–11.8)	11.2 (10.3–12.9)	14.15 (12.6–15.5)	<0.001
SVCF mL/kg/min, median (IQR)	97 (73–117)	99 (84–122)	112 (89–138)	112 (94–131)	114 (85–129)	<0.001
PDAF mL/kg/min, median (IQR)	37 (0–130)	54 (0–174)	66 (0–244)	0 (0–20)	0 (0–0)	<0.001
PBF mL/kg/min, median (IQR)	338 (263–411)	436 (340–560)	471 (370–621)	383 (323–453)	339 (280–447)	<0.001
PDA mm, median (IQR)	1.2 (0–1.9)	1 (0–1.89)	1.4 (0–2)	0 (0–1.08)	0 (0–0)	<0.001

*LVO* left ventricular output, *LVOd* the inner diameter of the ascending aorta, *PBF* pulmonary blood flow, *PDAF* patent ductus arteriosus flow, *PMA* postmenstrual age, *IQR* 1–3 interquartiles, *RVO* right ventricular outflow, *RVOd* diameter of the right ventricular outflow, *SVCd* superior vena cava diameter, *SVCF* superior vena cava flow, *VTI* velocity-time integral.

### The ductal blood flow

**Fig. 3:**
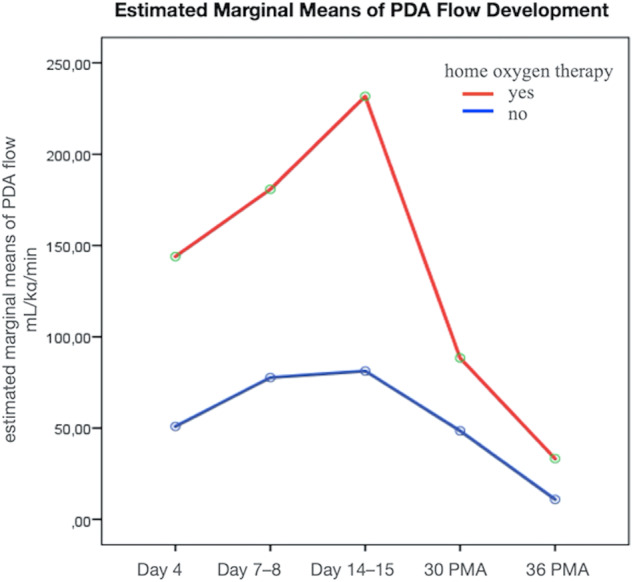
Development of PDA flow in infants with and without need for home oxygen therapy, a general repeated–measures model. Both groups showed the inverted U-shaped dynamics. However, the patterns displayed disparity.

For the entire sample, ductal blood flow changes significantly during postnatal development, and the effect size of the changes is large (*p* < 0.001, *η*^2^ = 0.186, Greenhouse-Geisser). Notably, the dynamics follow the inverted U-shape with an additional linear trend (both quadratic and linear contrasts: *p* < 0.001, both large effect size, *η*^2^ = 0.288 and *η*^2^ = 0.289 respectively). Both groups (with/ without need for home oxygen therapy) showed the inverted U-shaped dynamics (Fig. 3). However, the patterns displayed quantitative variations (*p* = 0.042, *η*^2^ = 0.052, small effect size). This disparity was especially evident in their linear trends (*p* = 0.042, *η*^2^ = 0.089, medium effect size) and overall flow over time (*p* = 0.006, *η*^2^ = 0.150, medium effect size). Infants embarked on the study with pre-existing disparities in conditions. Observable differences in flow were detected between the two groups at both the first (“Day 4”) and third (“Day 14–15”) measurements. Significantly, the disparity during the initial measurement remained robust even after Bonferroni correction (Mann–Whitney test, *p* = 0.006 and *p* = 0.031 respectively). For a general linear repeated-measures model, we tested a number of confounding factors: Gestational age, birth weight, Apgar score at the first and fifth minutes, antenatal steroids. Despite all these potential confounders, the differing linear trends between groups retained their significance (*p* = 0.017, *η*^2^ = 0.123, medium effect size). Table 5 shows an overview of CBF development in the group with and without future need for home oxygen therapy.

**Table 5:** An overview of CBF development and PDA diameter in the group with and without future need for home oxygen therapy.

	Day 4		Day 7–8		Day 14–15		30 weeks’ PMA		36 weeks’ PMA		*P* value
Home O2, *n*	Yes 27	No 31	Yes 27	No 32	Yes 26	No 32	Yes 27	No 30	Yes 28	No 28	
LVO median (IQR)	**397** (295–460)	305 (223–346)	**437** (372–584)	340 (280–421)	**551** (368–709)	351 (294–496)	343 (315–423)	365 (286–432)	300 (263–352)	327 (270–366)	
RVO median (IQR)	270 (225–338)	290 (229–321)	354 (273–410)	380 (314–447)	351 (269–429)	362 (309–427)	378 (325–469)	328 (287–389)	352 (273–422)	311 (257–411)	
SVC flow median (IQR)	93 (80–117)	101 (72–121)	99 (84–115)	99 (84–123)	113 (89–153)	112 (90–128)	114 (94–138)	109 (91–127)	111 (81–131)	114 (90–129)	
PDA flow* median (IQR)	**126** (2–213)	20 (0–58)	**106** (0–309)	5 (0–118)	**158** (0–376)	14 (0–150)	0 (0–13)	0 (0–29)	0 (0–0)	0 (0–0)	0.042
Pulmonary flow median (IQR)	**393** (285–530)	296 (258–377)	417 (362–657)	439 (338–520)	572 (361–664)	446 (374–538)	403 (330–536)	376 (293–424)	364 (280–505)	319 (279–415)	
PDA diameter median (IQR)	1.5 (0.2–2.0)	0.7 (0–1.8)	1.8 (0–2.0)	0.5 (0–1.5)	1.95 (0–2.2)	0.94 (0–1.75)	0 (0–0.65)	0 (0–1.4)	0 (0–0)	0 (0–0)	

*PDA flow = LVO –2.7 × SVC flow, a general linear repeated-measures model. Values highlighted in bold show the most striking differences.

*LVO* left ventricular output, *PDA* patent ductus arteriosus, *PMA* postmenstrual age, *IQR* 1–3 interquartiles, *RVO* right ventricular outflow, *SVC* superior vena cava.

## Discussion

Our data can provide a basic overview of the postnatal development of CBF values (LVO, RVO and SVC flow) and derived flow values (PDA flow and pulmonary blood flow) from the 4th day of life to 36 weeks of PMA in the most immature neonates born before 26 weeks of gestation. Each measured CBF value had a characteristic development based on postnatal adaptation. These data also show a unique clear association between high calculated PDA flow and unfavorable respiratory outcome (as home oxygen dependence).

A large proportion of studied preterm population born before 26 weeks’ gestation was significantly affected by a prolonged left-to-right ductal shunt. We observed PDA ≥ 1.5 mm in 48% of our infants in the first two weeks. Semberova et al. reported an even higher percentage of PDA in their study because they observed a predominantly untreated population^12^. Clyman et al. also observed a high incidence of large-to-moderate PDA in the younger group of infants at <25 weeks of gestation: 85% in untreated and 24% in treated infants at 7–8 days^13^. We also partially treated (in 40%) the observed neonates (15% prophylactically with indomethacin and 25% therapeutically with iboprufen). The low success rate of the late treatment in our study group can be explained by the extreme immaturity and probably by the late start of treatment in the 2nd to 3rd week of life.

Since blood flow in the PDA is directed from the aorta to the pulmonary artery, after pulmonary resistance has decreased, high pulmonary flow leads to high left heart preload, resulting in high stroke volume and, thus, high LVO. In infants with hemodynamically significant PDA (hsPDA), dilatation of the left heart, atrium, and ventricle is typically observed, sometimes even accompanied by mitral regurgitation. A clearly visible flow through the pulmonary veins is also characteristic. As a manifestation of the steal phenomenon, negative diastolic flow can be observed in the renal and mesenteric arteries or even in the cerebral arteries. The heart may also attempt to compensate for this condition by increasing contractility and cardiac output^14^. Therefore, high preload and a compensatory increase in contractility lead to the high LVO that characterizes hsPDA. This is consistent with the high LVO values obtained during the first three measurements when PDA was prevalent in our population. Most left-to-right shunt effects disappeared before week 30 of the PMA, so we observed decreasing LVO during weeks 30 and 36 of the PMA. We studied only stabilized children after the transient phase, and consequently, did not observe a low LVO (<150 mL/kg/min) after day 4, which is consistent with previously published studies^15^.

The greatest increase in RVO was observed between days 4 and day 7–8 in both the groups. This can be explained by the still decreasing pulmonary vascular resistance in the first days of life. A large PDA delays the normal physiological decrease in pulmonary vascular resistance. High pulmonary flow associated with pulmonary congestion increases pulmonary vascular resistance. Evans, Kluckow, and others also observed that PDA has a negative effect on RVO^15^. Another unfavorable factor may be decreased compliance of the right heart due to immaturity and, to some extent, possibly an extremely dilated left heart^16^.

Low SVC flow (< 45 mL/kg/min) is occasionally described in the early transitional period^4,17,18^; however, we did not encounter it in our population beyond the third day of life. The SVC flow was the most stable of the observed central flows. It increases slowly gradually with age, likely reflecting myocardial and vascular maturation. This stability of cardiac input and maintenance of cerebral flow play an important role in ensuring adequate output in various disease states, including PDA shunting^1^. In the other studies from the transitional period^17^ and beyond that^19^ the SVC flow was relatively stable and did not correlate with LVO or RVO.

Assuming that the SVC remains stable, the LVO alone can serve as a good indicator of the significance of a left-to-right ductal shunt. The LVO is the sum of the systemic flow and the left-to-right flow. Measurement of LVO can be done very quickly and easily and is suitable for practical clinical use at the bedside to assess the significance of a PDA in normal cardiopulmonary compensated infants. El-Khuffash et al. used LVO:celiac artery flow or LVO:SVC as a marker for the hemodynamic significance of PDA^20^. In certain circumstances, when SVC flow is variable, estimation of PDA flow may be inaccurate.

Our measured vessel diameters were consistent with previously published weight-corrected vessel diameters reported by de Waal et al.^21^ Sloot et al. observed CBF values in a stable population at less than 32 weeks of gestation. In contrast to our population, Sloot et al. observed a higher RVO and a lower LVO, and no significant difference between days 7 and 14^5^. This can be explained by the fact that our population was significantly more immature and burdened by a left-to-right ductal shunt; hence, a higher LVO prevailed. The values in the Sloot study may be closer to those in our group without home oxygen therapy, which was less burdened by a left-to-right shunt. In other studies, significantly lower LVO values, and higher RVO values were observed than those in our study. The explanation for these differences, in addition to differences in methodology, immaturity, prolonged PDA and postnatal age, is the severity of RDS and the need for artificial ventilation all of which affect CBF^22^. Bischoff et al. also measured lower values of LVO in their retrospective study. Smaller diameters of the aorta and pulmonary arteries were observed^19^. This resulted in overall lower LVO and RVO values. The SVC flow were similar to that in our measurements. Several studies have reported mean RVO values ranging from 202 mL/kg/min to 450 mL/kg/min^21^. The values we observed were the closest to those of Evans and Kluckow, probably because of the more similar methodology^15^, although we observed a narrower range.

The lungs of premature infants born at < 26 weeks of gestation are in the canalicular stage. The respiratory tree is growing, and vessels along the airways are being formed. In the canalicular stage, only 11% of the combined ventricular output reaches the lungs, corresponding to approximately 30–50 mL/kg/min^23^. In the postnatal group of the most immature infants, we calculated a lung flow of 300–600 mL/kg/min, i.e., ten times higher. How do immature pulmonary capillaries tolerate the tenfold increase in flow that occurs within a few hours of preterm birth? Vascular endothelial cells (ECs) form a specific microenvironment called the vascular niche, in which capillary ECs interact with other resident cells to regulate development, homeostasis, and regeneration and are important for signaling processes^24^. Pulmonary ECs regulate the postnatal pulmonary circulation by secreting various vasoactive molecules, including NO. ECs may act as mechanosensors that influence angiogenesis. Dysregulation may lead to arrest of secondary septation as observed in patient with BPD. Chronic pulmonary overflow delays normal pulmonary vascular maturation, smooth muscle retention^25^ and can result in damage to ECs. Therefore maladapted or injured ECs may contribute to the pathological development of lung tissue in BPD. This close relationship between capillaries and lung development^26^ may explain the association observed between high PDA flow and the need for home oxygen therapy.

For the outcome, we chose the need for home oxygen therapy rather than the degree of BPD. The reason for this choice was the inconsistency in the different BPD classifications and the imperfection of BPD prediction for future quality of life and the development of chronic lung disease.

The observed association between high PDA flow and the future need for home oxygen therapy should not be confused with causality. Lower weight, higher immaturity, fewer antenatal steroids, twins, low Apgar scores, higher need for intubation in the delivery room, and longer tracheal intubation are risk factors for developing BPD and were more pronounced in our group requiring home oxygen therapy. Clyman et al. also found the association between BPD and hsPDA depended on the length of intubation^27^. PDA treatment can have serious side effects and negatively affect other functions in immature neonatal organs^28,29^. However, further studies are required to address this issue.

In our analysis, the PDA diameter did not differ statistically significantly between the groups with and without home oxygen therapy. Is the diameter alone the best guide for selective treatment of PDA, although it has been so used in many studies? There are a number of studies in which treatment was targeted according to PDA diameter but had no clear positive effect on infant outcomes. The treatment only affected PDA diameter, but not respiratory or neurodevelopmental outcomes^30,31^. This could be due to several biases such as having information on only the PDA diameter without knowledge of PDA flow and duration, the lack of information on the effect of treatment on diameter, and the potential for inaccurate classification of infants solely based on diameter. Our study did not focus primarily on this problem. We anticipate that in the future, PDA flow or LVO together with PDA diameter will be more accurate predictors of adverse effects on lung function than PDA diameter itself. The hemodynamic significance of PDA depends not only on the size of the PDA, but also on the magnitude of the shunt and the ability of the myocardium to adapt to the extra shunt volume^32^.

When interpreting functional echocardiography, it should be noted that all ultrasound measurements are subject to internal errors, ranging from approximately 10% for intraobserver variability to 15–20% for interobserver variability^33^. Echocardiographic functional examination with CBF measurement requires precise determination of vessel diameters. A small deviation results in a large margin of error in the estimated flow through the vessel, because the diameter is squared in the formula used to calculate the cardiac output. Correctly displaying the SVC diameter is technically the most demanding task. The modified method from the suprasternal view may allow for a more accurate estimation of SVC flow^19^. The use of the published corrected vessel diameters can reduce these errors^21^. Reproducibility of echocardiographic measurements correlated well with invasive measurements^34^. Echocardiographic assessment of both LVO and SVC flow has been previously validated using cardiac magnetic resonance imaging^19^. Ficial et al. showed a strong correlation between echocardiographic assessment of LVO, and a poor correlation of SVC flow with phase-contrast magnetic resonance imaging assessment^35^. Another limitation of our study is that these data were not fully normative because the group included unwell infants. In addition, the left-to-right atrial shunt was not accurately identified. The other limitations are associated with omitting deceased participants from the analysis. This exclusion may limit the generalizability of the study findings and introduce bias into the results. The positive aspects of this study include its unique and extremely immature population, long-term follow-up, and prospective nature.

The measurement of central blood flow values is a suitable non-invasive method for monitoring the development of postnatal hemodynamic adaptation in immature neonatal populations. It allows monitoring of the PDA shunt and more accurate quantification of its significance, as well as monitoring the extent and duration of high pulmonary flow. Further prospective studies could use a more accurate estimate of the significance of the left-to-right ductal shunt, through LVO or PDA flow, to allow more precise targeting of PDA treatment or intervention aimed at preventing BPD in the highly vulnerable immature population. The association of PDA flow with an unfavorable respiratory outcome gives us an incentive to individualize treatment in extremely immature children.

## Data Availability

Anonymized data are publicly available to all researchers who wish to use them for non-commercial purposes without violating participant confidentiality: 10.5281/zenodo.10155249.
