# Publisher Correction: Association between patent ductus arteriosus flow and home oxygen therapy in extremely preterm infants

**DOI:** 10.1038/s41390-024-03177-5

**Published:** 2024-04-30

**Authors:** Jana Termerova, Ales A. Kubena, Karel Liska, Viktor Tomek, Richard Plavka

**Affiliations:** 1 Department of Gynecology, Obstetrics and Neonatology, First Faculty of Medicine, Charles University and General University Hospital in Prague, Prague, Czech Republic; 2 Institute of Medical Biochemistry and Laboratory Diagnostics, First Faculty of Medicine, Charles University and General University Hospital in Prague, Prague, Czech Republic; 3 Children’s Heart Center, Second Faculty of Medicine, Charles University and Motol University Hospital in Prague, Prague, Czech Republic

Correction to: *Pediatric Research* 10.1038/s41390-024-03120-8, published online 07 March 2024

Due to typesetting mistake, the units for the ‘estimated marginal means of PDA flow mL/kg/min’ in Figure 3 were given incorrectly.

Figure 3 in the original article:
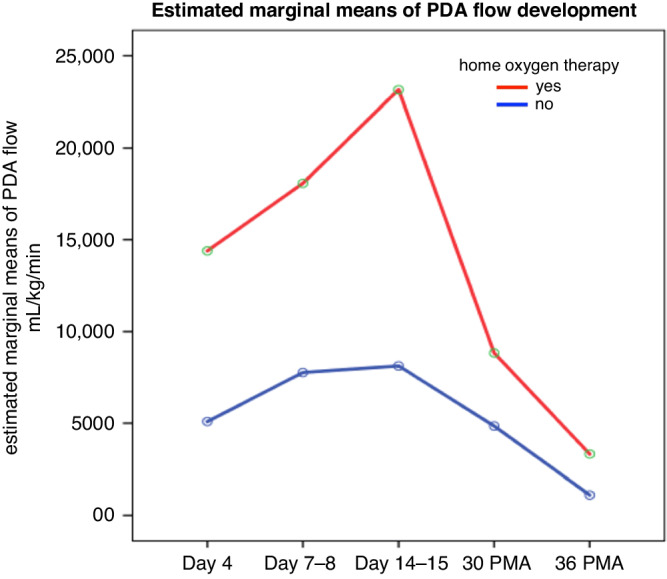


Corrected Figure 3 in the updated article:
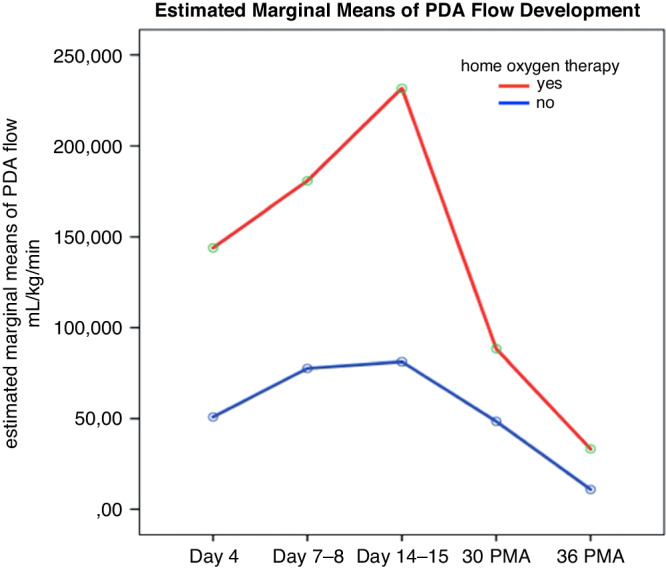


The original article has been corrected.

